# Brain Structural and Functional Alterations Specific to Low Sleep Efficiency in Major Depressive Disorder

**DOI:** 10.3389/fnins.2020.00050

**Published:** 2020-01-31

**Authors:** Ying Yang, Dao-min Zhu, Cun Zhang, Yu Zhang, Chunli Wang, Biao Zhang, Wenming Zhao, Jiajia Zhu, Yongqiang Yu

**Affiliations:** ^1^Department of Radiology, The First Affiliated Hospital of Anhui Medical University, Hefei, China; ^2^Department of Sleep Disorders, Affiliated Psychological Hospital of Anhui Medical University, Hefei, China; ^3^Hefei Fourth People’s Hospital, Hefei, China; ^4^Anhui Mental Health Center, Hefei, China; ^5^Department of Clinical Laboratory, The First Affiliated Hospital of Anhui Medical University, Hefei, China

**Keywords:** brain, major depressive disorder, magnetic resonance imaging, polysomnography, sleep efficiency

## Abstract

**Background:**

Sleep disturbance is common in patients with major depressive disorder (MDD), but the exploration of its neural underpinnings is limited by subjective sleep measurement and single-modality neuroimaging analyses.

**Methods:**

Ninety six patients with MDD underwent polysomnography examinations and multi-modal magnetic resonance imaging (MRI) scans. According to sleep efficiency, patients were subdivided into well-matched normal sleep efficiency (NSE, *N* = 42; 14 men; aged 43 ± 10 years) and low sleep efficiency (LSE, *N* = 54; 23 men; aged 45 ± 12 years) groups. Inter-group differences in brain structure and function were examined by applying voxel-based morphometry (VBM), regional homogeneity (ReHo) and functional connectivity strength (FCS), and tract-based spatial statistics (TBSS) approaches to structural, functional, and diffusion MRI data, respectively.

**Results:**

There was no significant difference in gray matter volume (GMV) between the NSE and LSE groups. Compared with the NSE group, the LSE group showed increased axial diffusivity in the left superior and posterior corona radiata, and left posterior limb and retrolenticular part of internal capsule. In addition, the LSE group exhibited decreased ReHo in the bilateral lingual gyri and right postcentral gyrus yet increased FCS in the left angular gyrus relative to the NSE group. Moreover, validation analyses revealed that these results remained after adjusting for the medication effect.

**Conclusion:**

Our data indicate that preserved gray matter morphology, impaired white matter integrity, and decreased local synchronization degree yet increased FCS are specific to low SE in MDD patients. These findings of disassociation between structural and functional alterations might provide insights into the neural mechanisms of sleep disturbance in depression.

## Introduction

Major depressive disorder (MDD) is a prevalent psychiatric illness affecting more than 300 million people of all ages worldwide, and it is characterized by abnormalities in mood, cognition, neurovegetative function, and psychomotor activity (American Psychiatric Association, 2013; World Health Organization [WHO], 2017; Frankish et al., 2018). It is well established that sleep problems (e.g., difficulty in falling asleep, difficulty in maintaining asleep or poor sleep quality, and difficulty in returning to sleep after early morning awakenings) are common complaints in MDD patients and up to 90% of the patients may experience sleep problems at some points of their illness trajectory (Tsuno et al., 2005). Thus, identification of relevant features specific to sleep disturbance in MDD is important for precise clinical diagnosis, treatment, and research.

Several predisposing factors including genetical determined dysfuntions (e.g., adenosine, gamma-aminobutyric acid receptor polymorphisms, and Clock genes), neurobiological mechanisms (e.g., homeostatic and circadian dysregulation), and personality characteristics (e.g., emotional suppression, maladaptive perfectionism, and neuroticsm) might contribute to the etiology and pathophysiology of sleep disturbance in MDD (Tsuno et al., 2005; Riemann et al., 2019). Importantly, previous neuroimaging studies using [^18^F]fluorodeoxyglucose positron emission tomography (PET) techniques have reported a reduction in brain glucose metabolism changes during the wake-sleep cycle in depressed patients relative to healthy subjects (Germain et al., 2004; Nofzinger et al., 2004, 2005).

The advent of magnetic resonance imaging (MRI) techniques and numerous methodological advances have provided a safe, non-invasive, and easily repeated neuroimaging avenue to exploring brain structural and functional changes in patients with psychiatric disorders (Agarwal et al., 2010; Lui et al., 2016). More recently, an increasing number of MRI research has emerged to investigate the neural mechanisms underlying various sleep disturbances in MDD. For example, Yu et al. (2018) found that patients with co-occurring depression and insomnia [Hamilton Rating Scale for Depression (HAMD) sleep subscale score > 3] showed smaller brain volume in the right orbitofrontal cortex when compared to patients with lower insomnia/depression. Liu et al. (2018) reported patients with MDD and high insomnia (HAMD sleep subscale score ≥ 4) had increased amplitude of low-frequency fluctuations in the right inferior frontal gyrus/anterior insula when compared with patients with MDD and low insomnia (HAMD sleep subscale score ≤ 3). Tao et al. (2018) found that functional connectivity alterations of the suprachiasmatic nuclei were associated with early-wakening symptom (HAMD-6 score = 1 or 2 for waking early in the morning) in MDD. McKinnon et al. (2018) suggested that sleep disturbance [the total score of Pittsburgh Sleep Quality Index (PSQI) > 5] in older people with a lifetime history of depression was related to increased functional connectivity in the default mode network. Notably, these studies measured sleep quality using subjective scales (e.g., PSQI) rather than objective tools [e.g., polysomnography (PSG)] and the neuroimaging analyses only focused on a single MRI modality.

PSG-derived sleep efficiency (SE), defined as the ratio of total sleep time (TST) to time in bed (TIB), is the most commonly used quantitative measure to objectively evaluate sleep quality (Akerstedt et al., 1994; Jung et al., 2017). A higher value of SE indicates more consolidated sleep because SE considers all sleep continuity variables (sleep latency, final wake time, and wake after sleep onset) in its calculation (Akerstedt et al., 1994; Hasler and Troxel, 2010). For MDD patients, depressive symptoms such as fatigue, anhedonia, and physical inactivity may lead to more time spent sleeping throughout the night and day, but less time obtaining efficient, restful, and uninterrupted sleep (Patel et al., 2006; Lacruz et al., 2016). SE can better capture this core feature of sleep disturbance in MDD. In light of evidence that SE decreases with age in healthy individuals (Ohayon et al., 2004) and SE is greater than 90% in normal young and middle-aged adults (Carrier et al., 2001; Gaudreau et al., 2001), a cutoff point of 90% was used to differentiate MDD patients with normal and low SE in the current study. With respect to MRI techniques, we adopted structural, functional and diffusion MRI (sMRI, fMRI, and dMRI) to assess brain structure and function. Specifically, gray matter volume (GMV) was measured by applying voxel-based morphometry (VBM) approach to sMRI (Ashburner and Friston, 2000). For resting-state fMRI, regional homogeneity (ReHo) and functional connectivity strength (FCS) approaches were employed to assess gray matter function. ReHo measures the degree of functional synchronization between a given voxel and its neighboring voxels (Zang et al., 2004; Liu et al., 2012). FCS is a graph theory measure that evaluates functional connectivity of each voxel with all other voxels across the whole brain (Tomasi and Volkow, 2010; Zuo et al., 2012; Liu et al., 2015; Zhu et al., 2017). In addition, tract-based spatial statistics (TBSS) analysis of diffusion MRI allows us to examine changes in multiple diffusion parameters, which reflect white matter integrity (Smith et al., 2006).

Collectively, the objective of this study was to test brain structural and functional differences between MDD patients with normal and low SE using a combined method of PSG and multi-modal MRI. We hypothesized that MDD patients with low SE would exhibit more severe brain aberrations than those with normal SE.

## Materials and Methods

### Participants

A total of 96 right-handed MDD patients with age between 18 and 60 years old were enrolled in this study. Patients were recruited from Department of Sleep Disorders, Affiliated Psychological Hospital of Anhui Medical University. The diagnosis of MDD was determined by two well-trained clinical psychiatrists according to the International Classification of Diseases (ICD-10). Exclusion criteria were (1) the presence of other psychiatric disorders such as schizophrenia, bipolar disorder, substance-induced mood disorder, anxiety disorders, substance abuse or dependence; (2) a history of significant neurological or physical diseases; (3) a history of head injury with loss of consciousness; (4) pregnancy or any contraindications for MRI. 24-item HAMD and 14-item Hamilton Rating Scale for Anxiety (HAMA) were used to assess the severity of depression and anxiety symptoms (Hamilton, 1967; Thompson, 2015). Subjective sleep quality and excessive daytime sleepiness were evaluated by using PSQI and Epworth Sleepiness Scale (ESS), respectively. All patients were receiving their regular antidepressant medications, either with selective serotonin reuptake inhibitors (SSRIs), serotonin norepinephrine reuptake inhibitors (SNRIs) or noradrenergic and specific serotonergic antidepressant (NaSSA). This study was approved by the Ethics Committee of The First Affiliated Hospital of Anhui Medical University and written informed consent was obtained from each participant.

### Polysomnography Examination

Full overnight PSG monitoring was performed on the patients using an Embla N7000 instrument (New York, NY, United States) according to the American Academy of Sleep Medicine (AASM) rules (Berry et al., 2012). All participants had to refrain from alcohol, caffeine and tea during the recording days. Neurophysiological variables [electroencephalogram (EEG), electrooculogram (EOG), chin electromyogram (EMG), and lower extremity movement], cardiorespiratory variables [electrocardiogram (ECG), thoracic and abdominal movements, oxygen saturation (SpO2), oronasal flow], and other variables such as body position were recorded. The signals were automatically recorded and subsequently analyzed. In our design, the following variables were extracted: TIB, TST, sleep time in non-rapid eye movements (NREM), and rapid eye movements (REM) periods. TIB was defined as the time from “lights out” to “lights on.” TST was defined as the sum of sleep time in NREM and REM periods. SE was defined as the ratio of TST to TIB. According to the value of SE, MDD patients were further divided into normal sleep efficiency (NSE) group of 42 patients with SE ≥ 90% and low sleep efficiency (LSE) group of 54 patients with SE < 90%.

### Image Acquisition

All MDD patients underwent MRI scanning in the morning after PSG recordings. MRI scans were obtained using a 3.0-Tesla MR system (Discovery MR750w, General Electric, Milwaukee, WI, United States) with a 24-channel head coil. High-resolution 3D T1-weighted structural images were acquired using a brain volume (BRAVO) sequence with the following parameters: repetition time (TR) = 8.5 ms; echo time (TE) = 3.2 ms; inversion time (TI) = 450 ms; flip angle (FA) = 12°; field of view (FOV) = 256 mm × 256 mm; matrix = 256 × 256; slice thickness = 1 mm, no gap; 188 sagittal slices; and acquisition time = 296 s. Resting-state blood-oxygen-level-dependent (BOLD) data were acquired using a gradient-echo single-shot echo planar imaging (GRE-SS-EPI) sequence with the following parameters: TR = 2000 ms; TE = 30 ms; FA = 90°; FOV = 220 mm × 220 mm; matrix = 64 × 64; slice thickness = 3 mm, slice gap = 1 mm; 35 interleaved axial slices; 185 volumes; and acquisition time = 370 s. DTI data were acquired using a spin-echo single-shot echo planar imaging (SE-SS-EPI) sequence with the following parameters: TR = 10,000 ms; TE = 74 ms; FA = 90°; FOV = 256 mm × 256 mm; matrix = 128 × 128; slice thickness = 3 mm without gap; 50 axial slices; 64 diffusion gradient directions (*b* = 1000 s/mm^2^) plus five *b* = 0 reference images; and acquisition time = 700 s. Before the scanning, all subjects were instructed to keep their eyes closed, relax, move as little as possible, think of nothing in particular, and not fall asleep during the scans. During and after scanning, we asked subjects whether they had fallen asleep to confirm that none of them had done so. None of the participants were excluded for visually inspected imaging artifacts.

### Voxel-Based Morphometry Analysis

The 3D T1-weighted structural images were processed using the VBM8 toolbox^1^ in Statistical Parametric Mapping software (SPM8)^2^. First, all the structural images were visually inspected to screen for artifacts or gross anatomical abnormalities. Then, all the structural images were segmented into gray matter, white matter and cerebrospinal fluid using the standard segmentation model. After an initial affine registration of the gray matter concentration map into Montreal Neurological Institute (MNI) space, the gray matter concentration images were non-linearly warped using the diffeomorphic anatomical registration through the exponentiated Lie algebra (DARTEL) technique and then resampled to a voxel size of 1.5 mm × 1.5 mm × 1.5 mm. The GMV map was obtained by multiplying the gray matter concentration map by the non-linear determinants that were derived from the spatial normalization step. Finally, the resultant GMV images were smoothed with a 6 mm full-width at half-maximum (FWHM) Gaussian kernel. The aims of smoothing were (1) to improve the signal to noise ratio; (2) to make the imaging data satisfy a Gaussian distribution, which is the prerequisite for parametric statistics; and (3) to further overcome the alignment problem in the spatial normalization step.

### DTI Data Preprocessing

The DTI datasets were pre-processed with the FMRIB Software Library (FSL v5.0.9)^3^. First, eddy current distortion and head motion were corrected by registering the diffusion-weighted images to the first b0 image through the affine transformations. Second, the data were skull-stripped by using the FMRIB Brain Extraction Tool. Finally, diffusion parameters of fractional anisotropy (FA), axial diffusivity (AD), radial diffusivity (RD), and mean diffusivity (MD) were calculated by using the DTIFIT toolbox.

### Tract-Based Spatial Statistics Analysis

Voxel-wise statistical analyses of the diffusion parameters were performed by using TBSS pipeline (Smith et al., 2006). First, individual FA images were aligned to the MNI space by using FMRIB Non-linear Imaging Registration Tool. After transformation into the MNI space, mean FA image was created and thinned to generate a mean FA skeleton. Then, individual aligned FA, AD, RD, and MD images were projected onto this common skeleton.

### fMRI Data Preprocessing

Resting-state BOLD data were preprocessed using Data Processing and Analysis for Brain Imaging (DPABI)^4^ (Yan et al., 2016). The first 10 volumes for each participant were discarded to allow the signal to reach equilibrium and the participants to adapt to the scanning noise. The remaining volumes were corrected for the acquisition time delay between slices. Then, realignment was performed to correct the motion between time points. Head motion parameters were computed by estimating the translation in each direction and the angular rotation on each axis for each volume. All participants’ BOLD data were within the defined motion thresholds (i.e., translational or rotational motion parameters less than 2.5 mm or 2.5°). We also calculated frame-wise displacement (FD), which indexes the volume-to-volume changes in head position. Several nuisance covariates (the linear drift, the estimated motion parameters based on the Friston-24 model, the spike volumes with FD > 0.5, the white matter signal, and the cerebrospinal fluid signal) were regressed out from the data. The datasets were then band-pass filtered using a frequency range of 0.01–0.1 Hz. In the normalization step, individual structural images were firstly co-registered with the mean functional image; then the transformed structural images were segmented and normalized to the MNI space using the DARTEL technique. Finally, each filtered functional volume was spatially normalized to MNI space using the deformation parameters estimated during the above step and resampled into a 3 mm cubic voxel.

### Regional Homogeneity Analysis

The ReHo calculation procedure was the same as that reported in previous studies (Zang et al., 2004). ReHo can be used to measure the degree of local regional neural activity coherence. In short, it was calculated as the Kendall’s coefficient of concordance (or Kendall’s W) of the time course of a given voxel with those of its nearest neighbors (26 voxels). For the purpose of standardization, the ReHo value of each voxel was divided by the global mean ReHo value. Finally, the resulting ReHo images were spatially smoothed with a 6 mm FWHM Gaussian kernel.

### Functional Connectivity Strength Analysis

We computed Pearson’s correlation coefficients between the BOLD time courses of all pairs of voxels and obtained a whole gray matter functional connectivity matrix for each participant (Tomasi and Volkow, 2010; Zuo et al., 2012; Liu et al., 2015; Zhu et al., 2017). For a given voxel, FCS was computed as the sum of positive functional connectivity above a threshold of 0.6 between that voxel and all other voxels within the whole gray matter. Then, we normalized the FCS value of each voxel by dividing it by the global mean FCS value. Finally, the resultant FCS maps were spatially smoothed with a 6 mm FWHM Gaussian kernel.

### Statistical Analyses

Demographic variables [age, years of education, body mass index (BMI), total intracranial volume (TIV), and FD], clinical parameters (onset age, illness duration, HAMD, HAMA, PSQI, and ESS), and PSG parameters (TIB, TST, NREM duration, REM duration, and SE) were compared between the NSE and LSE groups using two sample *t*-tests. Group difference in gender was tested by using Pearson Chi-square test. Pearson correlation analyses were used to examine the associations between SE and self-report sleep measures of PSQI and ESS. These statistical analyses were performed by using the SPSS 23.0 software package (SPSS, Chicago, β).

Voxel-based comparisons of GMV, ReHo, and FCS between the NSE and LSE groups were conducted by using the parametric two sample *t*-tests in the SPM8 software. Multiple comparisons were corrected using a cluster-level family-wise error (FWE) method, resulting in a cluster defining threshold of *P* = 0.001 and a corrected cluster significance of *P* < 0.05. For TBSS analyses of four DTI parameters (FA, AD, RD and MD), the non-parametric permutation testing (permutation number = 5000) and threshold-free cluster enhancement (TFCE) in the FSL software were used for statistical inference. The FWE method was also used to correct for multiple comparisons with a corrected significance threshold of *P* < 0.05. If a measure exhibited a significant between-group difference in a cluster, the mean value within this cluster was extracted for subsequent region of interest (ROI)-based analysis. To validate the group comparison results, Pearson correlation analyses were also used to test the relationship between SE and ROI mean values.

In addition, since previous studies have indicated that antidepressant medication may affect brain structure and function in MDD (Dusi et al., 2015; Brakowski et al., 2017), we considered antidepressant types (SSRIs, SNRIs, and NaSSA) as a categorical variable and then repeated the ROI-based inter-group comparisons in GMV, ReHo, FCS, and DTI parameters by using a general linear model with antidepressant types as a nuisance covariate to exclude its potential influence.

## Results

### Demographic, Clinical, and PSG Characteristics

Demographic, Clinical and PSG data of the sample are listed in Table 1. For the demographic and clinical data, the NSE and LSE groups did not differ in age (two-sample *t*-test, *t* = −0.849, *P* = 0.398), education (*t* = −0.240, *P* = 0.811), BMI (*t* = −0.885, *P* = 0.379), TIV (*t* = −1.621, *P* = 0.108), FD (*t* = 1.189, *P* = 0.238), onset age (*t* = −1.341, *P* = 0.183), duration of illness (*t* = 0.954, *P* = 0.343), HAMD (*t* = −0.335, *P* = 0.739), HAMA (*t* = −0.814, *P* = 0.417), PSQI (*t* = −0.162, *P* = 0.872), ESS (*t* = −0.608, *P* = 0.545) and sex (Chi-square test, χ^2^ = 0.855, *P* = 0.355). For the PSG data, although the two groups did not differ in TIB (*t* = −1.135, *P* = 0.259), the LSE group had decreased TST (*t* = 5.811, *P* < 0.001), NREM duration (*t* = 4.121, *P* < 0.001), REM duration (*t* = 3.075, *P* = 0.003), and SE (*t* = 13.135, *P* < 0.001) relative to the NSE group. In addition, Pearson correlation analyses revealed that there were no significant correlations between SE and self-report sleep measures of PSQI (*r* = −0.001, *P* = 0.990) and ESS (*r* = −0.025, *P* = 0.810).

**TABLE 1:** Demographic, clinical, and PSG characteristics.

Characteristics	MDD		Statistics	*P-*value
	NSE	LSE		
Number of subjects	42	54	–	–
**Demographic and Clinical data**				
Age (yeas)	42.8 ± 10.3	44.7 ± 11.7	*t* = −0.849	0.398^b^
Education (years)	8.7 ± 3.5	8.9 ± 3.7	*t* = −0.240	0.811^b^
BMI (kg/m^2^)	22.9 ± 3.8	23.5 ± 3.6	*t* = −0.885	0.379^b^
TIV (cm^3^)	1333.7 ± 140.9	1382.8 ± 152.1	*t* = −1.621	0.108^b^
FD (mm)	0.16 ± 0.11	0.13 ± 0.12	*t* = 1.189	0.238^b^
Onset age (years)	36.6 ± 11.2	39.9 ± 12.4	*t* = −1.341	0.183^b^
Duration of illness (months)	75.4 ± 92.3	59.4 ± 65.5	*t* = 0.954	0.343^b^
HAMD	26.7 ± 12.6	27.6 ± 12.4	*t* = −0.335	0.739^b^
HAMA	17.8 ± 8.2	19.1 ± 8.1	*t* = −0.814	0.417^b^
PSQI	12.5 ± 5.1	12.6 ± 5.2	*t* = −0.162	0.872^b^
ESS	5.8 ± 5.6	6.4 ± 5.3	*t* = −0.608	0.545^b^
Gender (female/male)	28/14	31/23	χ^2^ = 0.855	0.355^a^
**Antidepressant Medication (Number Of Patients)**				
SSRIs	31	35	–	–
SNRIs	9	16	–	–
NaSSA	2	3	–	–
**PSG data**				
TIB (min)	511.6 ± 59.4	524.4 ± 50.9	*t* = −1.135	0.259^b^
TST (min)	480.6 ± 57.3	416.2 ± 51.1	*t* = 5.811	<0.001^b^
NREM duration (min)	412.1 ± 54.6	366.0 ± 54.2	*t* = 4.121	<0.001^b^
REM duration (min)	68.5 ± 29.5	50.2 ± 28.6	*t* = 3.075	0.003^b^
SE (%)	93.9 ± 2.3	79.5 ± 7.6	*t* = 13.135	<0.001^b^

**The data are shown as the mean ± SD. PSG, polysomnography; MDD, major depressive disorder; NSE, normal sleep efficiency; LSE, low sleep efficiency; BMI, body mass index; TIV, total intracranial volume; FD, frame-wise displacement; SSRIs, selective serotonin reuptake inhibitors; SNRIs, serotonin norepinephrine reuptake inhibitors; NaSSA, noradrenergic and specific serotonergic antidepressant; HAMD, Hamilton Rating Scale for Depression; HAMA, Hamilton Rating Scale for Anxiety; PSQI, Pittsburgh Sleep Quality Index; ESS, Epworth Sleepiness Scale; TIB, total time in bed; TST, total sleep time; NREM, non-rapid eye movements; REM, rapid eye movements; SE, sleep efficiency.^*a*^The P-value was obtained by Pearson Chi-square test.^*b*^The P-values were obtained by two-sample t-tests.**

### Intergroup Comparison in GMV

For the VBM analysis, we found that there was no significant difference in GMV between the NSE and LSE groups (*P* > 0.05, cluster-level FWE-corrected).

### Intergroup Differences in Diffusion Parameters

**FIGURE 1:**
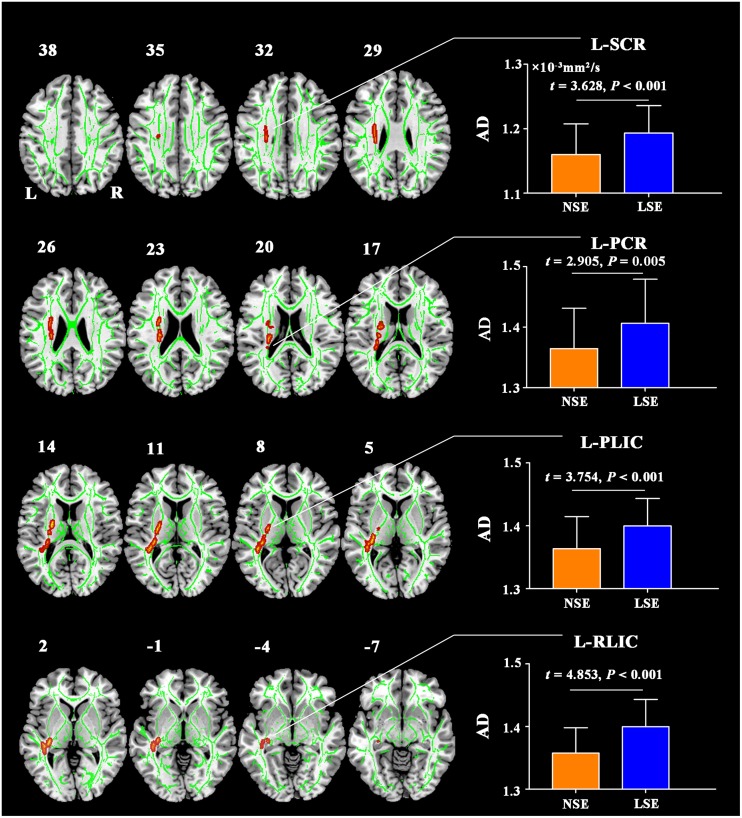
AD differences between the NSE and LSE groups. The numbers indicate the z-coordinates in the MNI space. Error bars indicate the standard deviation. AD, axial diffusivity; FA, fractional anisotropy; MNI, Montreal Neurological Institute; SCR, superior corona radiata; PCR, posterior corona radiata; PLIC, posterior limb of internal capsule; RLIC, retrolenticular part of internal capsule; NSE, normal sleep efficiency; LSE, low sleep efficiency; L, left; R, right.

Compared to the NSE group, the LSE group showed higher AD in the left superior corona radiata (SCR), left posterior corona radiata (PCR), left posterior limb of internal capsule (PLIC), and left retrolenticular part of internal capsule (RLIC) (*P* < 0.05, TFCE-FWE-corrected) (Figure 1). Further ROI-based analyses validated the TBSS comparison results (Figure 1); moreover, the significant inter-group differences in AD of the left SCR (*F* = 12.641, *P* = 0.001), left PCR (*F* = 8.261, *P* = 0.005), left PLIC (*F* = 14.974, *P* < 0.001), and left RLIC (*F* = 22.606, *P* < 0.001) were preserved after adjustment for the medication effect. However, there were no significant inter-group differences in other diffusion parameters including FA, RD, and MD (*P* > 0.05, TFCE-FWE-corrected).

### Intergroup Differences in ReHo

**FIGURE 2:**
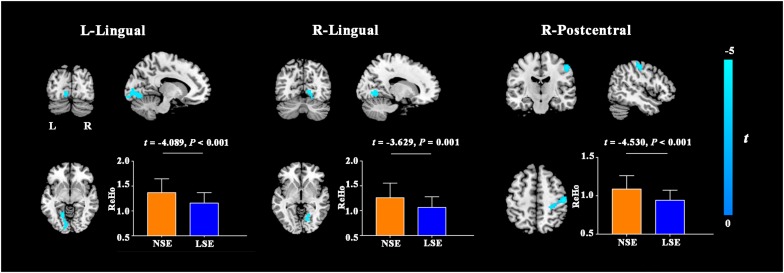
ReHo differences between the NSE and LSE groups. Error bars indicate the standard deviation. ReHo, regional homogeneity; NSE, normal sleep efficiency; LSE, low sleep efficiency; L, left; R, right.

Compared to the NSE group, the LSE group exhibited lower ReHo in the left lingual gyrus (cluster size = 152 voxels; peak MNI coordinates: x/y/z = −18/−60/−6; peak *t* = −4.2), right lingual gyrus (cluster size = 68 voxels; peak MNI coordinates: x/y/z = 21/−60/−9; peak *t* = −3.8) and right postcentral gyrus (cluster size = 179 voxels; peak MNI coordinates: x/y/z = 51/−18/51; peak *t* = −4.8) (*P* < 0.05, cluster-level FWE-corrected) (Figure 2). Further ROI-based analyses validated the voxel-based comparison results (Figure 2); moreover, the significant inter-group differences in ReHo of the left lingual gyrus (*F* = 16.727, *P* < 0.001), right lingual gyrus (*F* = 13.013, *P* = 0.001) and right postcentral gyrus (*F* = 20.558, *P* < 0.001) remained after controlling for the medication effect.

### Intergroup Differences in FCS

**FIGURE 3:**
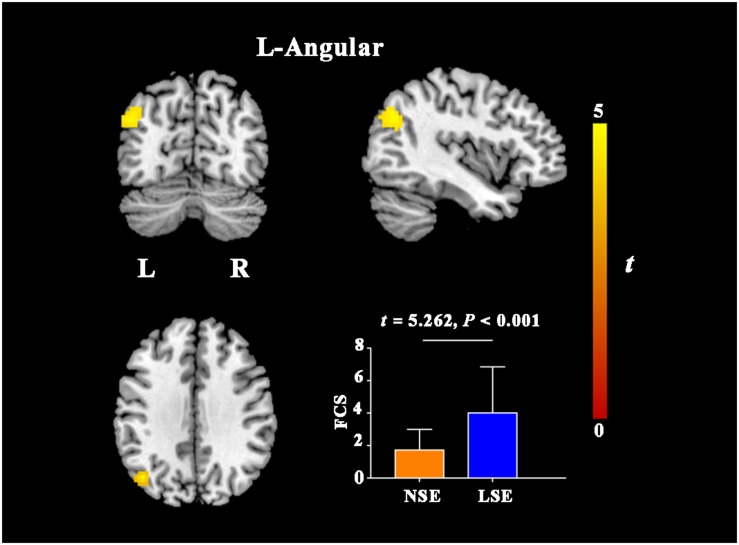
FCS differences between the NSE and LSE groups. Error bar indicates the standard deviation. FCS, functional connectivity strength; NSE, normal sleep efficiency; LSE, low sleep efficiency; L, left; R, right.

Compared with the NSE group, the LSE group presented with higher FCS in the left angular gyrus (cluster size = 101 voxels; peak MNI coordinates: x/y/z = −45/−69/33; peak *t* = 4.6) (*P* < 0.05, cluster-level FWE-corrected) (Figure 3). Further ROI-based analyses validated the voxel-based comparison results (Figure 3); moreover, the inter-group difference in FCS of the left angular gyrus was still significant after adjusting for the medication effect (*F* = 22.362, *P* < 0.001).

### Correlations Between SE and Neuroimaging Parameters

We also validated the group comparison results by conducting correlation analyses between SE and ROI mean values. There were significantly negative correlations between SE and AD in the L-PLIC (*r* = −0.204, *P* = 0.046) and L-RLIC (*r* = −0.357, *P* < 0.001), and marginally negative correlations between SE and AD values in the L-SCR (*r* = −0.194, *P* = 0.059) and L-PCR (*r* = −0.180, *P* = 0.079) (Supplementary Figure S1). Additionally, there were significant positive correlations between SE and ReHo in the left lingual gyrus (*r* = 0.314, *P* = 0.002), right lingual gyrus (*r* = 0.319, *P* = 0.002) and right postcentral gyrus (*r* = 0.395, *P* < 0.001) (Supplementary Figure S2), as well as a significant negative correlation between SE and FCS in the left angular gyrus (*r* = −0.266, *P* = 0.009) (Supplementary Figure S3).

## Discussion

By using PSG to objectively assess sleep quality and multimodal MRI techniques to comprehensively measure brain properties, we conducted the first exploratory analyses to investigate the neural substrates of low SE in MDD. Three major findings were recorded in our study. First, there was no significant difference in GMV between the NSE and LSE groups. Second, the LSE group exhibited increased AD in the left SCR, PCR, PLIC, and RLIC compared with the NSE group. Third, MDD patients with low SE showed decreased ReHo in the bilateral lingual gyri and right postcentral gyrus, and increased FCS in the left angular gyrus. These findings suggest that relative preservation of gray matter morphology, impaired white matter integrity, and decreased local synchronization degree yet increased FCS may represent the characteristic brain changes associated with low SE in MDD.

Our current observation that GMV did not differ between MDD patients with normal and low SE seems to be in contradiction with a previous study highlighting that smaller GMV in the OFC played a critical role in the neuropathology of the comorbidity of insomnia and depression (Yu et al., 2018). Difference in the sleep quality measurement is a highly likely source of the result inconsistency: in the Yu et al. study, insomnia in MDD was determined by HAMD sleep subscale score > 3; in our design, SE in the MDD patients was derived from the PSG. Other potential sources include differences in patient characteristics (e.g., MDD sample heterogeneity in medication), sMRI data processing approaches (VBM vs. FreeSurfer), and statistical analysis methods (direct inter-group comparison vs. two-factor analysis based on a general linear model) (Yu et al., 2018).

Compared to those with normal SE, MDD patients with low SE exhibited increased AD in the corona radiata (SCR and PCR) and internal capsule (PLIC and RLIC), implying that impaired axonal integrity (Song et al., 2002) in these white matter fibers may be specific to sleep disturbance in depression. The corona radiata is composed of ascending and descending fibers that relay information to and from the cerebral cortex, and is functionally implicated in emotional and executive processing (Karababa et al., 2015; Stave et al., 2017). The internal capsule is an important component of the thalamo-cortical circuits, and therefore plays a pivotal role in the processing of sensory and motor function, development of depressed mood or suicide, and even the regulation of daily cycles of sleep and wakefulness (Jia et al., 2010; Emos and Agarwal, 2019). In previous studies, impaired white matter integrity in the corona radiata and internal capsule has been demonstrated in both depressed patients and patients with primary insomnia (Li et al., 2016; Jiang et al., 2017). Combined, our current findings, coupled with the prior evidence, support the notion that white matter integrity impairments in the corona radiata and internal capsule may partly account for the disturbance in wake-sleep cycles in MDD patients.

The findings of reduced ReHo in the lingual and postcentral gyri in MDD patients with low SE suggest that decreased local synchronization degree in brain regions related to visual and somatosensory processing might be implicated in sleep disturbance in depression. In agreement with our reports, a resting-state fMRI study on MDD patients documented that decreased functional connectivity of the suprachiasmatic nuclei with the lingual gyrus and calcarine sulcus was linked to early wakening symptom (Tao et al., 2018). A prior PET study revealed that after sleep deprivation, depressed patients showed alterations in relative cerebral glucose metabolism in the visual areas and these metabolism alterations were associated with changes in depressive symptom severity (Wu et al., 2008). In addition, previous large sample brain imaging studies have consistently found structural (i.e., lower surface area; Schmaal et al., 2017) and functional (i.e., decreased neural activity and functional connectivity; Xia et al., 2019) deficits in the visual and somatosensory areas in MDD patients. On the basis of these findings, it is reasonable to assume that decreased local synchronization degree in the sensory system might cause visual and somatosensory processing dysfunctions, which in turn result in low SE in MDD. The underlying mechanism may be that impairment in sustained sensory processing of environmental stimuli may lead to a disruption of circadian rhythm and hamper the ability to initiate or maintain sleep.

In this study, we also found increased FCS in the angular gyrus [a core region of the default mode network (DMN)] in MDD patients with low SE, indicating the role of functional connectivity increase of the DMN in depression-associated sleep disturbance. Similarly, using ROI-to-ROI functional connectivity analyses, McKinnon and colleagues (McKinnon et al., 2018) showed that depression individuals with current sleep disturbance (PSQI > 5) had increased functional connectivity within the DMN relative to those without sleep disturbance (PSQI ≤ 5). The DMN is highly activated at rest but deactivated during goal-directed cognitive tasks, and thus is thought to be mainly engaged in internally directed cognitions such as emotional processing and self-referential activity (Buckner et al., 2008; Raichle, 2015). Moreover, there is evidence that the DMN also has prominent implications in sleep initiating and maintenance (Koenigs et al., 2010). In prior studies, depression and its related cognitive rumination have been attributed to increased DMN activity (Sheline et al., 2010; Zhang et al., 2011; Kuhn and Gallinat, 2013; Cheng et al., 2016). Of note, altered functional connectivity and regional neural activity in the DMN were also observed in patients with primary insomnia (Zhou et al., 2017; Yan et al., 2018). In normal subjects, there is a decrease in regional cerebral blood flow in the DMN regions during sleep (Maquet et al., 1996, 1997). However, a hyperarousal state (“over excitation of the nerves”) caused by acute stressors and cognitive rumination is considered a key component in all modern etiological models of insomnia disorder (Perlis et al., 1997; Buysse, 2013). Taken together with these previous findings, our data suggest that increased FCS of the DMN in MDD may be associated with the emergence of the hyperarousal state that in turn leads to the observed low SE in this disorder.

In our analyses, there were no significant differences in the PSQI and ESS between the NSE and LSE groups. There is evidence for a discrepancy between subjective and objective assessments of sleep in patients with MDD (Rezaie et al., 2018). Self-reported sleep rating can be affected by mood and memory biases (Weiss et al., 1973; Bliwise and Young, 2007). In patients with MDD, the perception of sleep is a function of depression severity, cognition, and actual sleep, rather than actual sleep alone (Tsuchiyama et al., 2003). Previous studies have demonstrated that depressed patients could not accurately estimate their sleep time and they are more likely to poorly recall aspects of sleep (Rotenberg et al., 2000). This may lead to some clinical reports of MDD patients complaining of poor sleep without any abnormal polysomnographic findings (Castro et al., 2013), which are in agreement with the current observation.

There are several limitations that should be acknowledged in this study. First, the sample size is fairly modest, which may limit the statistical power in detecting subtle brain alterations and uncovering potential depression-brain-sleep relationship. Furthermore, we cannot absolutely rule out the influence of antidepressant medication and illness duration on our results. However, the validation analyses showed that our results were not changed by medication and the NSE and LSE groups were well-matched in illness duration, which may alleviate concerns about these confounding factors. Future studies are warranted to test the reproducibility of our findings by using a larger sample of first-episode, medication-naive MDD patients. Second, we did not exclude some sleep disorders such as insomnia, obstructive sleep apnea and periodic leg movements in sleep, which may affect our interpretation. Future studies in MDD patients without any sleep disorders are required to validate our findings. Third, relative to hospitalized MDD patients, healthy subjects are more affected by the “first-night effect” of PSG, characterized by a disruption of sleep on the initial PSG recording compared to those from subsequent nights (Song et al., 2013). This may result in unreliable PSG variables in healthy subjects, so we did not include healthy controls in this study. In future studies, adding an adaptation night or alternative sleep measurement approaches such as actigraphy are potential methods for addressing the “first-night effect.” Finally, our cross-sectional design does not allow inference on causality. Longitudinal studies with intervention targeted toward improving SE in depression patients are needed to establish the direction of causality.

## Conclusion

In conclusion, this study systematically investigated brain structural and functional substrates of low SE in MDD using a combination of PSG and multi-modal MRI. Our data indicate that MDD patients with low SE have relatively preserved gray matter morphology but impaired white matter integrity in the corona radiata and internal capsule relative to patients with normal SE. In addition, decreased local synchronization degree in the sensory system yet increased FCS in the default mode network are specific to low SE in MDD patients. These findings of disassociation between structural and functional alterations might provide insights into the neural mechanisms of sleep disturbance in depression.

## Data Availability Statement

The datasets generated for this study are available on request to the corresponding author.

## Ethics Statement

The studies involving human participants were reviewed and approved by the Ethics Committee of The First Affiliated Hospital of Anhui Medical University. The patients/participants provided their written informed consent to participate in this study.

## Author Contributions

D-MZ, JZ, and YYu conceptualized and designed the study. YYa was responsible for conducting the analyses, preparing the first draft of the manuscript, and preparing the manuscript for submission. JZ was responsible for obtaining funding for the study, supervising the analyses, and editing drafts of the manuscript. WZ, YZ, CZ, and BZ were responsible for data collection and initial data preprocessing. All authors contributed to and approved the final manuscript.

## Conflict of Interest

The authors declare that the research was conducted in the absence of any commercial or financial relationships that could be construed as a potential conflict of interest.

## Footnotes

1 http://dbm.neuro.uni-jena.de/vbm.html

2 http://www.fil.ion.ucl.ac.uk/spm

3 www.fmrib.ox.ac.uk/fsl

4 http://rfmri.org/dpabi
