# Supplementary material for: Brain Structural and Functional Alterations Specific to Low Sleep Efficiency in Major Depressive Disorder
Source: Front Neurosci. 2020 Jan 31;14:50. doi: 10.3389/fnins.2020.00050 (PMC7005201; doi:10.3389/fnins.2020.00050)
Supplement: Supplementary file 1 [file Table_1.doc]

***Supplementary materials***


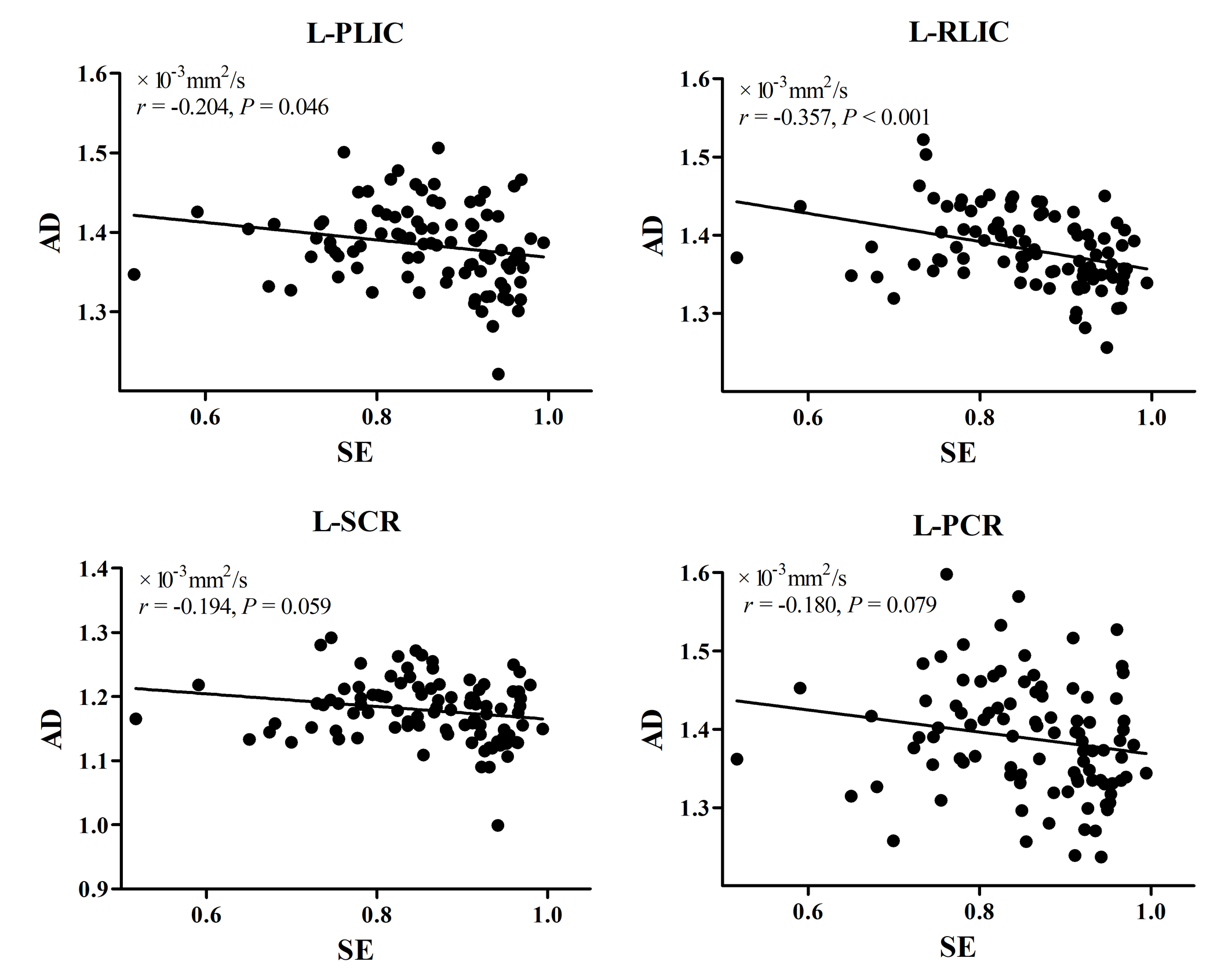


**Figure S1.** Scatter plots of the correlations between SE and AD. Abbreviations: AD, axial diffusivity; SE, sleep efficiency; SCR, superior corona radiata; PCR, posterior corona radiata; PLIC, posterior limb of internal capsule; RLIC, retrolenticular part of internal capsule; L, left; R, right.


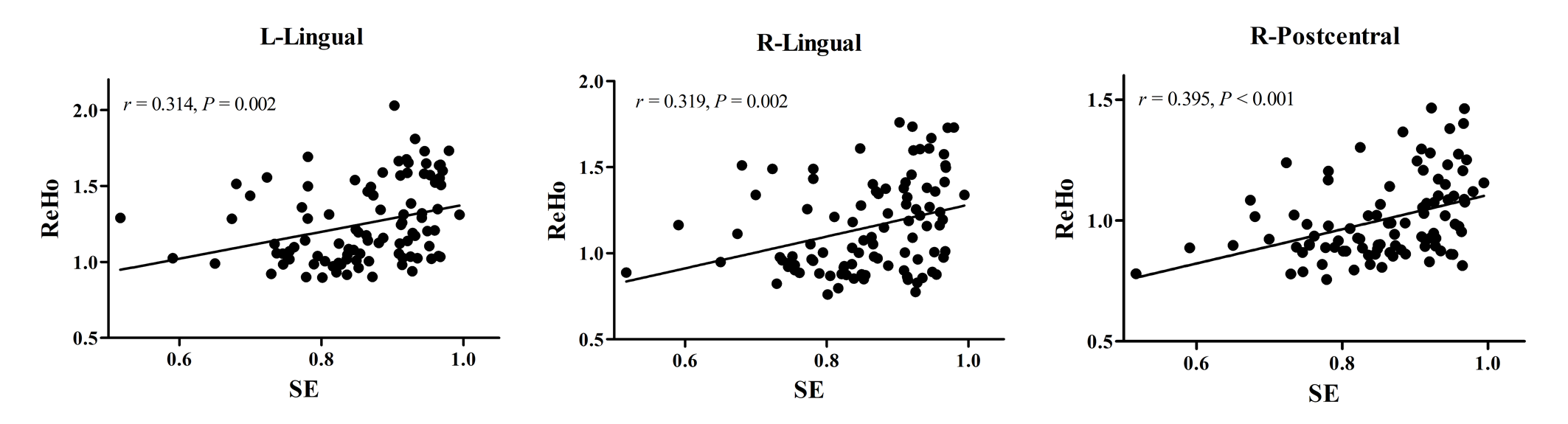


**Figure S2.** Scatter plots of the correlations between SE and ReHo. Abbreviations: ReHo, regional homogeneity; SE, sleep efficiency; L, left; R, right.

**
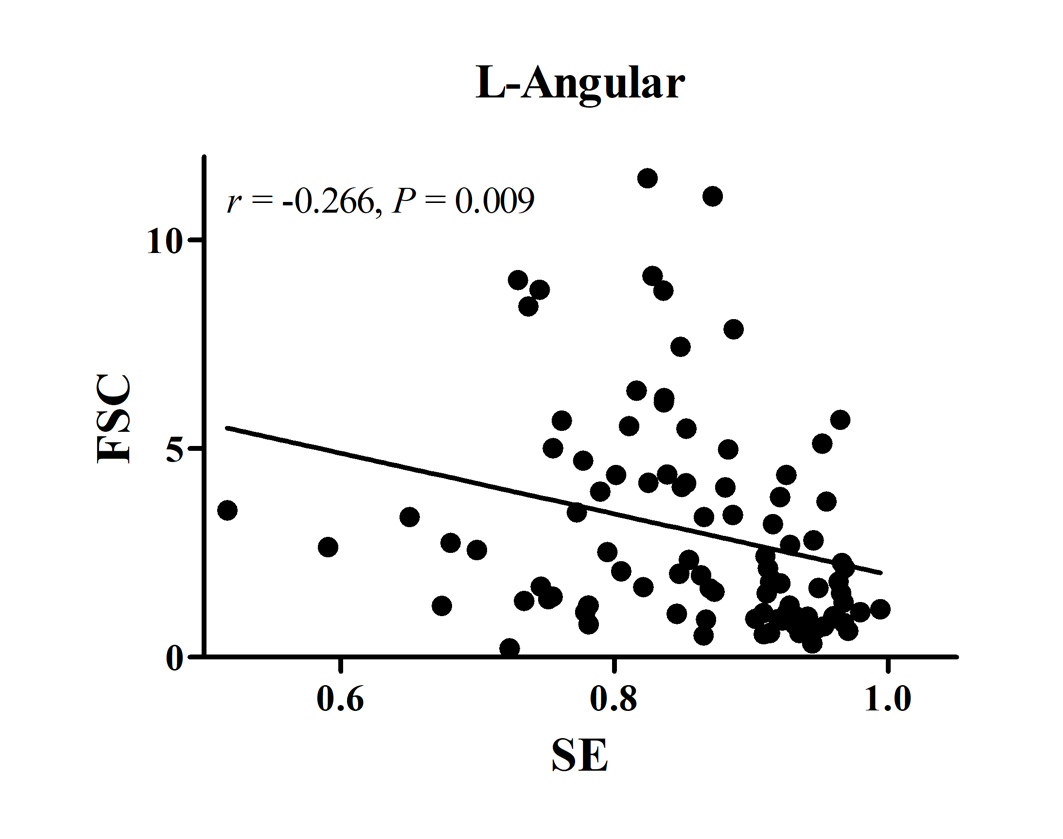
**

**Figure S3.** Scatter plots of the correlations between SE and FCS. Abbreviations: FCS, functional connectivity strength; SE, sleep efficiency; L, left; R, right.
